# Pseudomyogenic hemangioendothelioma mimicking widespread metastatic malignancy - An unusual presentation of a rare condition

**DOI:** 10.1016/j.jdcr.2025.01.034

**Published:** 2025-03-24

**Authors:** Raaisa Islam, Vivek Bhadri, Kenneth Wong, Karen Cheung

**Affiliations:** a The Skin Hospital, Sydney, New South Wales, Australia; b Faculty of Medicine and Health, University of Sydney, Sydney, New South Wales, Australia; c Chris O’Brien Lifehouse, Sydney, New South Wales, Australia; d Blacktown Dermatology, Sydney, New South Wales, Australia; e Douglas Hanly Moir, New South Wales, Australia

**Keywords:** case report, chemotherapy, dermatopathology, FDG PET, FOSB, malignancy, metastatic, mTOR inhibitor, oncology, pseudomyogenic hemangioendothelioma, rare condition, sirolimus

## Introduction

Pseudomyogenic hemangioendothelioma (PHE) is a rare intermediate-grade (rarely metastasizing) vascular neoplasm that commonly presents as distinct, solitary nodules on distal extremities. It can be challenging to differentiate from clinicopathologically similar conditions. This distinction is important as PHE usually has a more indolent course.^1^ Until its official inclusion in the World Health Organization’s Classification of Soft Tissue and Bone Tumors in 2013, various descriptions of PHE existed in the literature. Mirra et al first described *fibroma-like variant of epithelioid sarcoma* in 1992 without investigating vascular markers. Billings et al subsequently described this entity as *epithelioid-sarcoma-like hemangioendothelioma* in 2003 before Hornick and Fletcher coined *pseudomyogenic haemangioendothelioma* in 2011.^2^

PHE typically affects young adults aged 20-40 years-old with a predilection toward males in a ratio of 4.6:1. Approximately two-thirds of cases have multifocal lesions that can involve different tissue depths in the same anatomic region such as the dermis (31%), subcutis (20%), muscle (34%), and bone (14%). Lesions can vary in size from a few millimetres to a few centimetres. Cutaneous lesions are typically well-circumscribed red-brown firm nodules (occasionally ulcerated), papules and/or plaques, commonly distributed in the lower extremities associated with or without pain. Skeletal involvement typically presents as multifocal lytic lesions without periosteal reaction or bone destruction.^1^, ^2^, ^3^, ^4^, ^5^, ^6^, ^7^

We present an unusual case of PHE mimicking widespread metastatic malignancy.

## Case report

**Fig 1:**
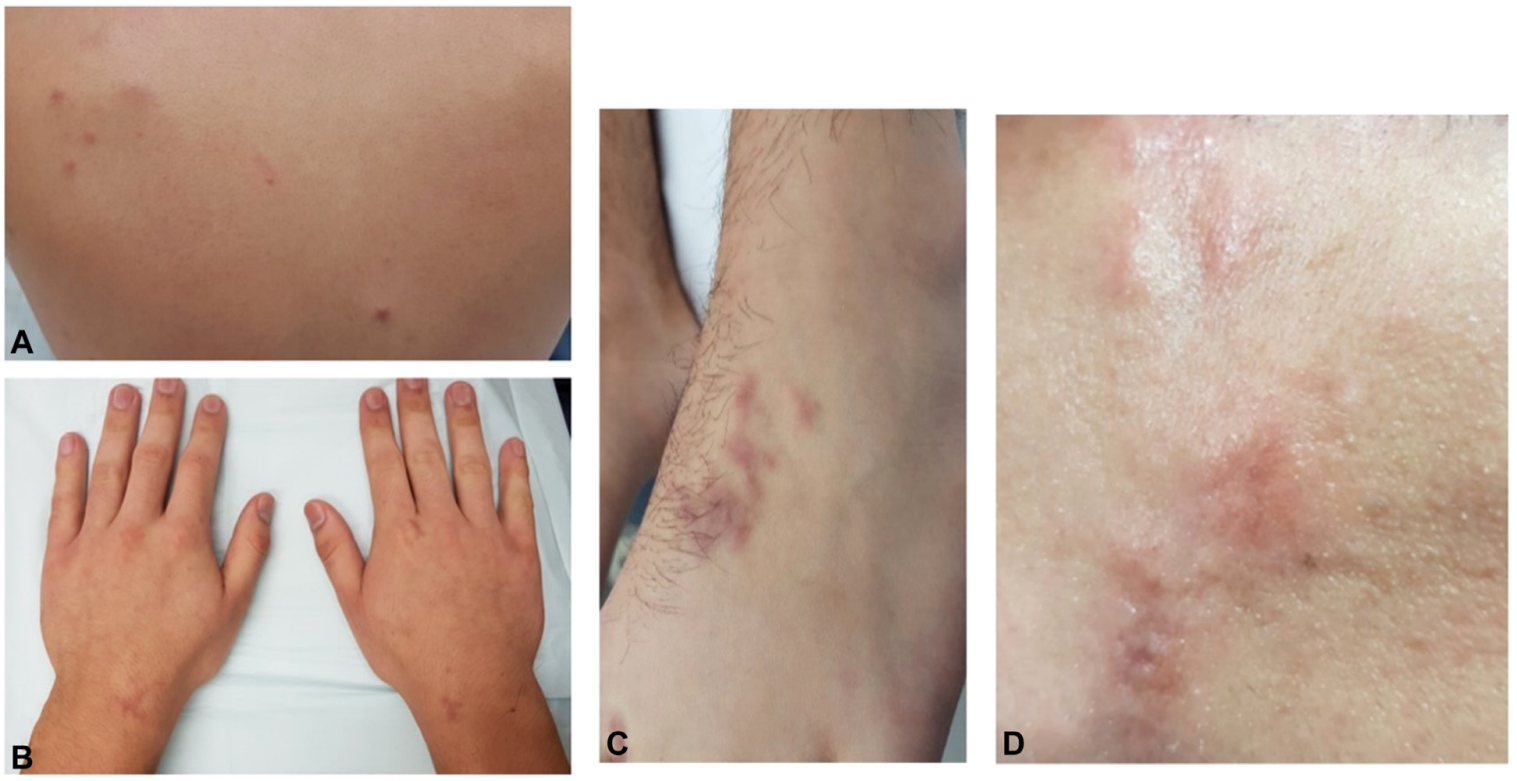
Widely distributed erythematous papules and nodules including the back (**A**), hands (**B**), left foot (**C**), and forehead (**D**, close-up view)

A 23-year-old male presented with an unresolving right arm pink papule which evolved over 1 month into multiple widespread, intermittently painful, erythematous papules, nodules, and plaques. He also reported localized myalgia, however no systemic symptoms. On examination, these were distributed in the upper and lower limbs, hands, feet, trunk, back, neck, and face (Fig 1). He had no significant past medical history, regular/recent medications, allergies, family history or recent exposure to pathogens. The clinical appearance of these lesions suggested infiltration of the dermis/epidermis, and considerations were made for soft tissue sarcomas, metastatic carcinomas, sarcoidosis, and cutaneous lymphoma. Routine blood tests were unremarkable apart from isolated elevation of bilirubin (likely reflective of Gilbert’s syndrome) and nonspecific mild elevation of lactate dehydrogenase.

**Fig 2:**
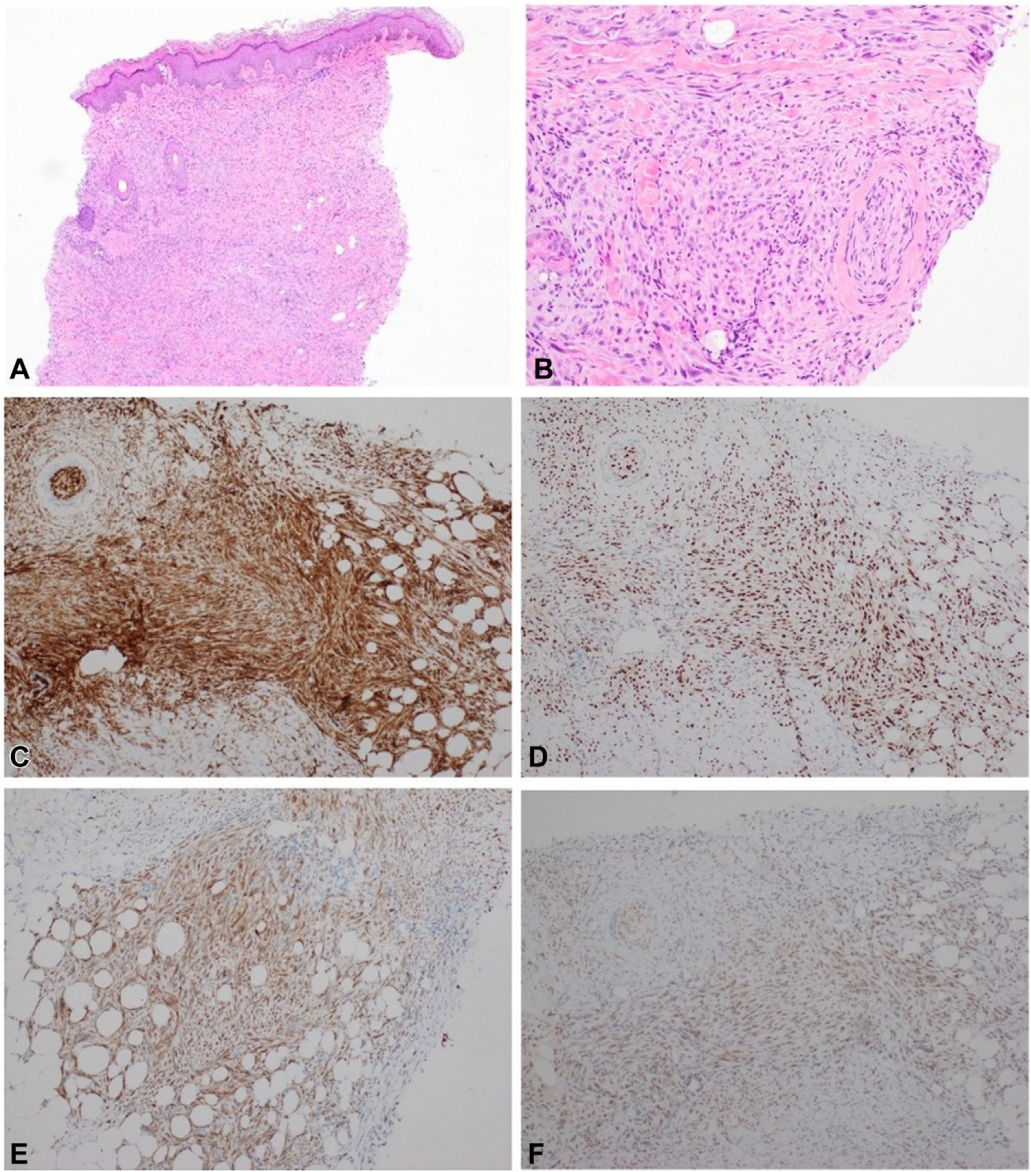
Histopathologic and immunohistochemical features of pseudomyogenic hemangioendothelioma. Intermediate power (**A**)∗ and high power (**B**)∗ hematoxylin & eosin stain (H&E) showing cells with prominent spindle shape with myoid or epithelioid sarcoma-like appearance. Tumor cells immunoreactive for CD31 (**C**), ERG (**D**), Pan Keratin (**E**), and INI 1 (**F**). ∗ White balance adjusted. *ERG*, Erythroblast transformation specific (ETS)-related gene.

Skin punch biopsies were taken from lesions involving the right arm and right knee. Both showed similar histologic features consistent with an atypical vascular tumor involving dermis and subcutis with aberrant keratin expression and a somewhat infiltrative architecture with foci of perineural and intravascular invasion. The cells had a prominent spindle shape with myoid or epithelioid sarcoma-like appearance (Fig 2). The histopathologic differentials included dermatofibrosarcoma, rhabdomyosarcoma, epithelioid sarcoma, epithelioid leiomyosarcoma, epithelioid angiosarcoma, or epithelioid hemangioendothelioma.

Immunostains were positive for cytokeratin (pan CK), CK7 and CK19, ERG, and CD31 (the latter 2 strongly positive) but were negative for EMA, CD34, and p63. INI1 nuclear expression was retained and FOS-B was positive. A diagnosis of PHE was confirmed by next generation sequencing molecular study showing ACTB-FOSB fusion.

**Fig 3:**
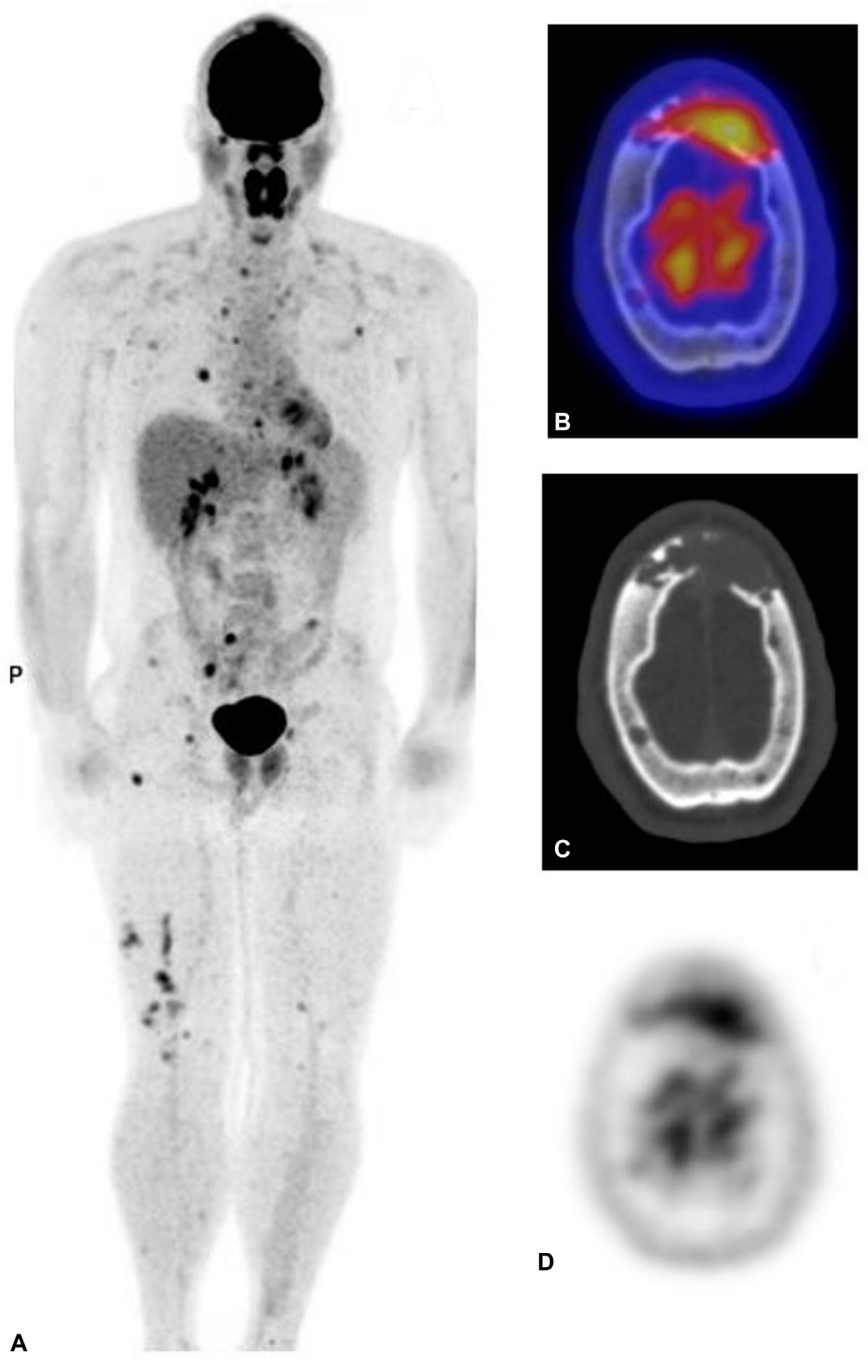
FDG PET scan. Maximum intensity projections showing distribution of hypermetabolic cutaneous, subcutaneous, intramuscular, and bony lesions (**A**). Large lytic lesion of the frontal bone with erosion of the inner and outer table of the calvarium seen on fused (**B**), CT (**C**), and PET (**D**) views. *FDG*, Fluorodeoxyglucose; *PET*, positron emission tomography; *CT*, computed tomography.

An F-18 fluorodeoxyglucose positron emission tomography scan identified multiple scattered hypermetabolic cutaneous, subcutaneous, and intramuscular lesions (Fig 3). Multiple destructive hypermetabolic lytic bone lesions were also seen in the skull (including large destructive frontal bone lesion with extension to the dura), ribs, mandibular condyle, multiple thoracic and lumbar vertebrae, pelvis, femoral head, and femora. No solid organ or lung lesions were seen.

They were initially commenced on betamethasone diproprionate 0.05% ointment to apply directly to lesions while awaiting biopsy results. Following confirmation of the diagnosis, the patient was also commenced on sirolimus and zoledronic acid. Progress positron emission tomography imaging showed fluctuating fluorodeoxyglucose uptake at most sites of disease with complete regression of only a few lesions. The patient has remained asymptomatic for over 2 years and continues on treatment with regular monitoring.

## Discussion

This case is a distinctly unusual clinical presentation of PHE mimicking widespread metastatic disease with widely distributed cutaneous lesions. Though the exact pathogenesis is not completely understood, PHE is identified cytogenetically by FOSB gene rearrangements. Most commonly, balanced t(7;19) (q22;q13) gene fusion causes a SERPINE1-FOSB gene fusion. However, a subset of alternative rearrangements produces ACTB-FOSB and WWTR1-FOSB fusion genes. FOSB is a member of the Fos family whose upregulation has a role in the tumourigenesis of multiple tumours. The SERPINE1-FOSB fusion gene leads to upregulation of FOSB which dimerises with Jun proteins to form AP-1 transcriptional factor complex. Both ACTB-FOSB and WWTR1-FOSB also code for chimeric transcription factors. ACTB-FOSB produces essentially similar clinical/pathologic appearances but is more commonly associated with solitary lesions, contrary to our case.^6^

FOSB overexpression is the most important immunohistochemical marker but should be interpreted in combination with histopathology as focal or diffuse nuclear staining can be seen in a variety of vascular and fibroblastic/myofibroblastic tumors.^6^^,^^8^ PHE typically has broad expression of cytokeratin (AE1/AE3). Endothelial differentiation may be implied in 50% of cases by CD31 expression, however there is a lack of CD34 expression which is not seen in other types of hemangioendothelioma. Nuclear FLI-1 and ERG is expressed in all cases. Unlike epithelioid sarcoma, nuclear INI1 is retained. Smooth muscle actin can be positive in a third of patients. EMA staining is typically weak.^1^

Presently, there is no established standard of care for PHE. For solitary lesions, local control measures have been described including surgical resection/local excision, curettage, cryotherapy, amputation, and postoperative radiotherapy. Local recurrence in adjacent soft tissues is described in one-fifth to over one-third of cases.^2^^,^^6^ For multifocal disease, several systemic therapies (sometimes in combination) have achieved variable rates of remission and disease stabilization. These have included cytotoxic chemotherapy, mammalian target of rapamycin inhibitors (eg sirolimus), tyrosine kinase inhibitors (eg telatinib and pazopanib), vascular endothelial growth factor inhibitors, and bone antiresorptive agents (such as bisphosphonates and denosumab). The mammalian target of rapamycin pathway is involved in the regulation of a range of cell function including angiogenesis, cell division and motility, and protein synthesis. Tyrosine kinase inhibitors may interfere with the self-regulation of fusion genes. PHE-related mortality is very uncommon. We found only 2 reported cases of mortality attributed to disseminate diseases (patients aged 19 and 86-years old) and an additional disputed case (82-year-old).^2^^,^^4^^,^^6^

This is a rare case of PHE mimicking metastatic malignancy. Establishing a diagnosis allows for the selection of appropriate therapies.

## Conflicts of interest

None disclosed.
